# Cave-dwelling phlebotomine sand flies (Diptera: Psychodidae: Phlebotominae) in Thailand: population composition and pathogen detection of *Bartonella* and *Trypanosoma*

**DOI:** 10.1186/s13071-024-06616-8

**Published:** 2024-12-19

**Authors:** Sakone Sunantaraporn, Puckavadee Somwang, Pathamet Khositharattanakool, Isaraporn Unchanam, Nattiya Saenchaiban, Wilai Wongkhut, Pinpinat Sanum, Thanapat Pataradool, Rungfar Boonserm, Jérôme Depaquit, Padet Siriyasatien

**Affiliations:** 1 Center of Excellence in Vector Biology and Vector-Borne Diseases, Department of Parasitology, Faculty of Medicine, Chulalongkorn University, Bangkok, Thailand; 2 School of Medicine, Mae Fah Luang University, Chiang Rai, Thailand; 3 Biomedical Technology Research Group for Vulnerable Populations, Mae Fah Luang University, Chiang Rai, Thailand; 4 The Office of Disease Prevention and Control 1 Chiang Mai, Chiang Mai, Thailand; 5 Bureau of Vector Borne Diseases, Department of Disease Control, Bangkok, Thailand; 6 Lampang Provincial Health Office, Lampang, Thailand; 7 Department of Parasitology, Faculty of Medicine, Bangkok, Thailand; 8 Faculté de Pharmacie , Université de Reims Champagne-Ardenne, SFR Cap Santé, EA7510 ESCAPE-USC ANSES VECPAR, Reims, France

**Keywords:** sand flies, *Trypanosoma* sp., *Bartonella* sp., *SSU* rRNA gene, *gltA* gene, Thailand

## Abstract

**Background:**

Leishmaniasis is an emerging vector-borne disease that occurs in Thailand. Although *Leishmania* (*Mundinia*) parasites, the causative agents of the disease have been identified, the vectors of the disease remain unidentified. In the present study, we collected sand flies from three caves located in endemic areas of leishmaniasis, including Lampang and Chiang Rai in northern Thailand, and Songkhla in southern Thailand.

**Methods:**

Female sand flies were identified on the basis of morphological characteristics and confirmed by cytochrome c oxidase subunit I (*COI*) sequencing. Sand fly DNA samples were screened for *Leishmania*, *Trypanosoma*, and *Bartonella* DNA by polymerase chain reaction (PCR) on the basis of the *ITS1* region of the ribosomal RNA (rRNA), *SSU* rRNA, and *gltA* genes, followed by phylogenetic relationships and haplotype diversity analysis.

**Results:**

A total of 557 sand flies were identified, comprising four genera (*Sergentomyia*, *Phlebotomus*, *Grassomyia*, and *Idiophlebotomus*) and 11 species. Molecular detection of pathogens demonstrated that *Leishmania* DNA was not detected. However, *Trypanosoma* DNA was detected in 11 samples of *Phlebotomus mascomai* from Lampang (7 for *T*. *noyesi*), *Se*. *anodontis* from Chiang Rai (1 each for *T*. *noyesi* and *Trypanosoma* sp.), and *Se*. *khawi* from Songkhla (2 for *Trypanosoma* sp.). *Bartonella* DNA was detected in 16 samples of *Se*. *anodontis* and *Se*. *barraudi* s.l. from Chiang Rai, *Se*. *anodontis* from Lampang, and *Se*. *khawi* from Songkhla. The novel *Bartonella* sp. detected in Thai sand flies was phylogenetically related to *Bartonella* sp. from bats. Genetic diversity analysis showed high haplotype diversity in both *Trypanosoma* parasites and *Bartonella* bacteria.

**Conclusions:**

The data from the present study indicate that phlebotomine sand flies could be potential vectors of zoonotic diseases caused by *Trypanosoma* sp. and *Bartonella* sp. To our knowledge, this is the first report of the natural infection of *Bartonella* associated with bats in Thailand, and the presence of *T*. *noyesi* and amphibian trypanosomes. However, further investigation is required to elucidate and enhance the understanding of potential vectors and transmission dynamics of pathogens in Thailand, particularly with regard to different seasonality, habitats, and host ranges.

**Graphical abstract:**

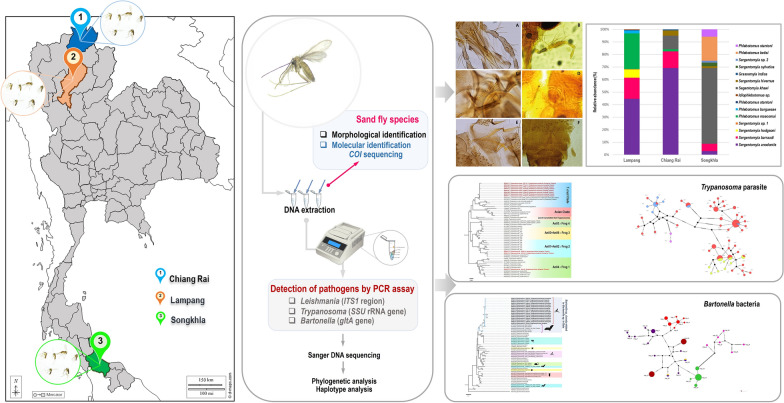

**Supplementary Information:**

The online version contains supplementary material available at 10.1186/s13071-024-06616-8.

## Background

Phlebotomine sand flies are small hematophagous insects belonging to the Psychodidae family [1]. These insects are generally found breeding in cool and moist areas, such as caves, animal shelters, hollow trees, soil in human habitats, and under stones [2, 3]. Sand flies can be found in urban and rural settings, especially in wild environments, including caves. Cave environments may provide excellent conditions for maintaining sand flies, owing to specific physical and environmental variables, such as temperature and humidity [2, 3]. Furthermore, the diversity and density of sand flies in caves can be higher than that found in other environments [3]. In Thailand, several reports of the composition of sand fly species have been investigated in endemic and nonendemic areas of leishmaniasis in northern and southern Thailand [4–7].

Sand flies are medically important vectors of several emerging and reemerging diseases distributed worldwide. Sand fly-borne pathogens have been reported in various sand fly species, including the *Leishmania* parasites, *Bartonella bacilliformis*, and some arboviruses of the genera *Phlebovirus*, *Vesiculovirus*, and *Orbivirus*, which can cause leishmaniasis, bartonellosis, and sand fly fever [8–10].

In Thailand, increasing cases of autochthonous leishmaniasis have been continuously reported in the southern and northern regions of the country [11–13]. Autochthonous leishmaniasis in Thailand is caused by two *Leishmania* of the subgenus *Mundinia*: *L*. *martiniquensis* [14, 15] and *L*. *orientalis* (previously named “*L*. *siamensis*”) [16]. Previous studies of the potential vector of *Leishmania* infections in endemic areas of Thailand have revealed that the DNA of *L*. *martiniquensis* was detected in three species of the *Sergentomyia* genus: *Sergentomyia khawi* [17, 18], *Se*. *barraudi*, and *Se*. *gemmea* [11, 19, 20] but the identification of the latter sand fly remains unsuccessful [5]. A recent survey has revealed the presence of *L*. *martiniquensis* in *Grassomyia indica* [21]. Furthermore, *L*. *orentalis* was detected in *Se*. *iyengari* [22] and *Se*. *khawi* within southern Thailand [18]. Sriwongpan et al. detected *L*. *orentalis* DNA in six samples of *Se*. *gemmea* and *L*. *martiniquensis* DNA in one sample of *Phlebotomus stantoni* collected from the Chiang Rai province of northern Thailand [23]. More interestingly, a study by Sunantaraporn et al. (2021) has demonstrated that biting midges, *Culicoides mahasarakhamense* are considered as a potential vector of *L*. *martiniquensis* in the Lamphun province of northern Thailand [24]. The recent investigation of Songumpai et al. (2022) revealed the first evidence of co-circulation of *L*. *martiniquensis* and *L*. *orientalis* in several *Culicoides* species: *C*. *peregrinus*, *C*. *oxystoma*, *C*. *mahasarakhamense*, and *C*. *huffi* collected near the house of a patient with leishmaniasis from the Songkhla province of southern Thailand [25]. However, the role of natural *L*. subgenus *Mundinia* infection vectors in Thailand remains unclear.

Several species of sand fly have been reported to be potential vectors of *Trypanosoma* parasites. These parasites were reported in endemic and nonendemic areas of leishmaniasis in southern Thailand [17, 21, 26, 27]. The most common *Trypanosoma* sp. reported in Thai sand flies was phylogenetically classified into anuran trypanosomes on the basis of small subunit ribosomal RNA (*SSU* rRNA) sequences. Some *Trypanosoma* sp. in sand flies were grouped by *T*. *noyesi* within the *T*. *cruzi* clade [17, 27], and other *Trypanosoma* sp. were closely related to *T*. *microti* and *T*. * kuseli*, species previously detected from rodents [26]. However, there is no evidence of *Trypanosoma* parasites that can be transmitted by sand flies, to humans or animals, in Thailand.

Apart from the parasites mentioned above, sand flies have been implicated as vectors of human bartonellosis caused by *Bartonella* bacterial infection [28]. *Bartonella* spp. is a gram-negative, fastidious intracellular and intra-erythrocytic alphaproteobacteria, which can infect human and vertebrate hosts [29]. Several blood-sucking insects are related to the transmission of *Bartonella* spp., including body lice (*Pediculus humanus humanus*) [30], cat fleas (*Ctenocephalides felis*) [31], cattle lice (*Haematopinus* sp.) [32], and the New World sand fly (*Pintomyia verrucarum*) [33]. Some species of ticks are classified as potential vectors of *Bartonella* species [34]. Sand flies were confirmed to be vectors of *B*. *bacilliformis* that causes Carrion’s disease or Oroya fever, and humans play an important role as accidental hosts infected by these bacteria [35]. In Thailand, *Bartonella* infection has been reported as a zoonotic pathogen in dogs, cats, rodents, and bats [36–39], and has also been detected in dog ticks, rat fleas, and cat fleas [40, 41]. However, information on the *Bartonella* bacteria in sand flies in Thailand has never been investigated. Therefore, the objective of this study was to screen for the presence of natural infection and genetic diversity in *Leishmania* and *Trypanosoma* parasites, as well as the first detection of *Bartonella* bacteria in cave-dwelling sand flies collected from the northern and southern regions of Thailand.

## Methods

### Study sites and sand fly collection

**Fig. 1:**
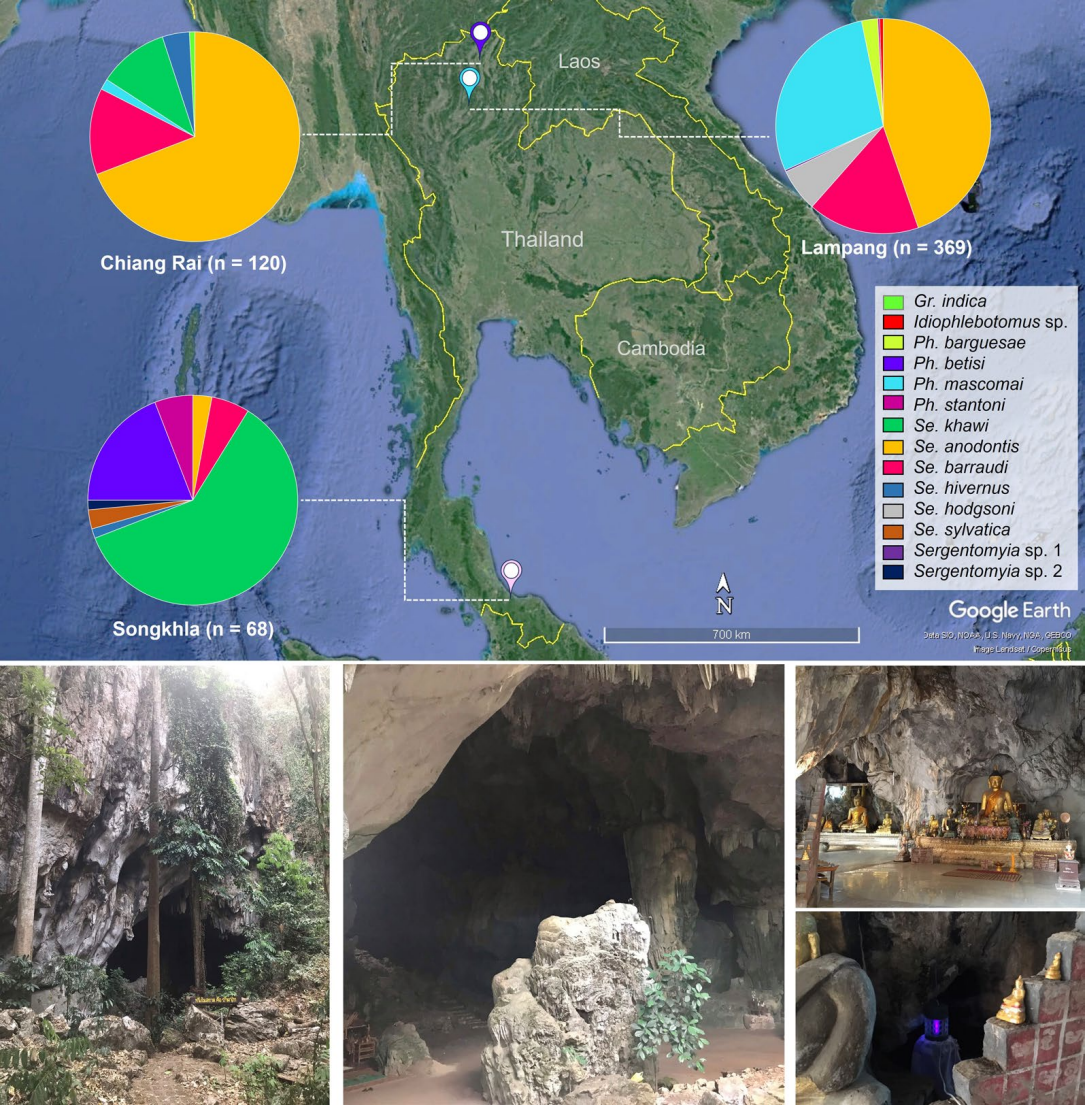
Map of Thailand, displaying three locations and species diversity of cave-dwelling sand flies collected from Chiang Rai, Lampang, and Songkhla provinces. Images obtained and modified from Google Earth Pro version 7.3.4.8248 (https://www.google.com/earth/about/)

Sand fly collections were carried out in limestone caves in three provinces, namely, Tham Phra (19°55′03″N 99°47′20″E) from Chiang Rai; Tham Pha Thai, located at Tham Pha Thai National Park (18°36′18.8″N 99°53′51.9″E) from Lampang in the northern region; and Tham Khao Rup Chang (6°43′50.9′′N 100°16′40.1′′E) in Songkhla province, southern Thailand (Fig. 1). The study sites were selected on the basis of tourist attractions, and were inhabited by many bats. Similarly, three provinces have reported several cases of autochthonous leishmaniasis caused by *L*. *martiniquensis* infection. The average distance between collection sites and leishmaniasis cases for each area was around 20–30 km. Sand flies were captured using miniature Center for Disease Control and Prevention (CDC) light traps (25W bulb) with ultraviolet (UV) light, in October 2020 and March 2021. The five traps were installed inside the caves at a height of approximately 50 cm above the ground and operated continuously for 12 h from 6.00 pm to 6.00 am the following morning for 2 nights per month. The insects caught in the collected bags were placed in a Petri dish and anesthetized in an ice box for 30 min. The female sand flies were sorted from other insects, preserved in 70% ethanol, and transported to the Center of Excellence in Vector Biology and Vector Born Diseases, department of parasitology, faculty of medicine, Chulalongkorn University.

### Morphological identification of sand fly species

Female sand flies were dissected under a stereomicroscope with single-use sterile needles in 0.9% sterile normal saline. The head and genitalia with the spermathecae were mounted onto a glass slide with Hoyer’s medium before being identified under a compound microscope according to the description of morphological keys [5, 26, 42]. The rest of the body parts (thorax, legs, wings, and abdomen) were placed in a sterilized 1.5 ml microcentrifuge tube and dried at room temperature for 15 min prior to DNA extraction.

### Genomic DNA extraction

Genomic DNA was extracted from individual female sand flies using a modified protocol, as detailed in the Genelute™ Mammalian Genomic DNA Miniprep Kit (Merck KGaA, Darmstadt, Germany) following the manufacturer’s instructions. ​In brief, the samples were lysed in 180 µl of lysis buffer (containing 20 µl of proteinase K) and crushed with a sterilized plastic pestle, then incubated at 56 °C overnight. DNA was eluted in 40 µl of prewarmed elution buffer. The quality of the DNA concentrations was measured with Nanodrop 2000c (Thermo-scientific, USA), and stored at −20 °C for further investigation.

### Detection of *Leishmania*, * Trypanosoma*, and *Bartonella* DNA in sand flies

To detect pathogens in sand flies, primer sets on the basis of three genes were selected: *Leishmania* sp. (*ITS1*), *Trypanosoma* sp. (*SSU* rRNA), and *Bartonella* sp. (*gltA*). These primer sets were chosen because of their high sensitivity and specificity, as well as their suitability for phylogenetic analysis. Conventional polymerase chain reaction (PCR) was carried out using primers LeF (5′-TCCGCCCGAAAGTTCACCGATA-3′) and LeR (5′-CCAAGTCATCCATCGCGACACG-3′) that targeted the *ITS1* region of the ribosomal RNA gene for the detection of *Leishmania* [43]. PCR reagents and amplification conditions were described by Sunantaraporn et al. [24]. For *Trypanosoma* detection, PCR amplification of the *Trypanosoma* sp. *SSU* rRNA gene was performed using primers TRY927F (5′-GAAACAAGAAACACGGGAG-3′) and TRY927R (5′-CTACTGGGCAGCTTGGA-3′) [44]. The PCR reaction and amplification were performed in accordance with those of Srisuton et al. [17]. The estimated product size for *Leishmania* and *Trypanosoma* was approximately 379 and 900 bp, respectively. The amplified products were separated on a 1.5% (W/V) agarose gel electrophoresis. The expected products were imaged with Quantity One Quantification Analysis Software Version 4.5.2 (Gel DocEQ System; Bio-Rad, Hercules, CA, USA), after staining with ethidium bromide.

The presence of *Bartonella* DNA in sand flies was tested in all DNA samples targeting the citrate synthase (*gltA*) gene, using the primers BhCS871p (5′-GGGGACCAGCTCATGGTGG-3′) and BhCS1137n (5′-AATGCAAAAAGAACAGTAAACA-3′) [45]. Conventional PCR was performed following the methods previously described by Promrangsee et al. [32]. The presence of an expected band of 379 bp was determined by 1.5% (W/V) agarose gel electrophoresis.

DNA extracted from *L*. *martiniquensis* promastigotes, *Trypanosoma evansi* DNA from blood-infected dogs, and *Bartonella* sp. detected from cattle lice DNA were used as positive controls and deionized distilled water was used as a negative control.

### *COI* amplification for sand fly species identification

To confirm the morphological identification of the sand fly, PCR was performed to amplify the partial mitochondrial cytochrome c oxidase subunit I (*COI*) gene using the invertebrate primers LepF (5′-ATTCAACCAATCATAAAGATATTGG-3′) and LepR (5′-AAACTTCTGGATGTCCAAAAAATCA-3′) [46]. PCR was performed under the following condition profiles: initial denaturation at 94 °C for 5 min, then 5 cycles at 94 °C for 30 s, annealing at 45 °C for 90 s, extension at 72 °C for 1 min, followed by 35 cycles of denaturation at 95 °C for 30 s, annealing at 51 °C for 90 s, extension at 72 °C for 1 min, and final extension at 72 °C for 10 min. PCR reactions were carried out in a total volume of 50 µL consisting of 10× PCR buffer, 25 mM of MgCl_2_ (Thermo Fisher Scientific, Waltman, MA, USA), 2.5 mM of dNTPs (Biotectrabbit, Berlin, Germany), 10 µM of each primer, 1 unit of *Taq* DNA polymerase (Thermo Fisher Scientific, Waltman, MA, USA), and 6 µL of DNA template. The expected size of the PCR product of the *COI* gene was determined by the presence of a band at approximately 700 bp. DNA extracted from *Culicoides peregrinus* was used as a positive control, and deionized distilled water was used as a negative control.

### DNA cloning and sanger nucleotide sequencing

All positive amplified pathogen detection products were inserted into the pGEM-T Easy Vector (Promega, Mandison, WI, USA) using DNA ligation kits (Promega, Mandison, WI, USA) following the manufacturer’s instructions. Then, 5 µL of DNA ligation were used to transform into *Escherichia coli* DH5α competent cell, and then the chimeric DNA was screened by blue–white colony selection and colony PCR assay. The white colonies suspected to contain the insert sequences were cultured and chimeric DNA was then extracted using the GeneAll^®^ Exprep™ Plasmid Purification Kit (GeneAll Biotechnology, co., ltd, Seoul, Korea) following the manufacturer’s instructions. The purified chimeric DNA was sequenced by the commercial service of Macrogen Inc., South Korea.

Direct sequencing was performed with the *COI* amplified products for the confirmation of sand fly species. The PCR product was purified using the QIAquick PCR purification kit (QIAGEN, Max-Volmer-StraBe4, Hilden, Germany) according to the manufacturer’s instructions. Direct sequencing was carried out using the corresponding forward and reverse primers for *COI* amplification by the Macrogen, Inc. commercial service.

### Phylogenetic construction, genetic diversity, and haplotype analysis

All derived nucleotide sequences were manually trimmed and edited prior to alignment using the ClustalW multiple alignment program in BioEdit Sequence Alignment Editor version 7.2.5 [47]. Consensus nucleotide sequences were compared with previously available sequences in the GenBank database using the Basic Local Alignment Search Tool (BLAST) (https://blast.ncbi.nlm.nih.gov/Blast.cgi). The phylogenetic trees were constructed using the maximum likelihood method on the basis of the lowest Bayesian information criterion (BIC) scores with 1000 bootstrap replicas on Molecular Evolutionary Genetics Analysis software version 11 (MEGA11) (https://www.megasoftware.net/) [48]. The ML tree was visualized using FigTree v.1.4.4 (http://tree.bio.ed.ac.uk/software/fig-tree). The intraspecific genetic divergence among sand fly species was calculated using the Kimura 2-parameter (K2P) model in MEGA11 [48].

The genetic diversity of *Trypanosoma* sp. and *Bartonella* sp. from the current study was evaluated in DnaSP version 6 (http://www.ub.edu/dnasp/) [49], to calculate the number of haplotypes (H), the number of polymorphic sites (S), the average number of nucleotide differences (k), haplotype diversity (Hd), and nucleotide diversity (*π*). To demonstrate the relationship between these pathogen haplotypes and their hosts, or other vector origins within Thailand, a TCS haplotype network [50] was generated using Population Analysis with Reticulate Trees software (PopART) version 1.7 (https://popart.maths.otago.ac.nz/) [51].

### Data analysis

The prevalence rate was calculated on the basis of the PCR-positive results by dividing the number of positive samples by the total number of samples collected in each area. The 95% confidence interval (CI) was employed. The relative abundance of sand fly populations was evaluated from the different sites (number of samples of species/total number of samples × 100). All data processing and descriptive statistics were performed in Microsoft Excel 2019 (Microsoft Corp., USA).

## Results

### Morphological and molecular identification of sand flies

A total of 557 female sand flies were trapped in limestone caves located in three provinces. Of these, 369 and 120 cave-dwelling female sand flies were collected in Lampang and Chiang Rai provinces in the northern region, and 68 female sand flies were collected from Songkhla province in southern Thailand. The present study identified all sand flies within four genera: *Sergentomyia*, *Phlebotomus*, *Idiophlebotomus*, and *Grassomyia*. On the basis of morphological identification, 11 sand fly species were identified as *Se*. *anodontis*, *Se*. *barraudi* s.l., *Se*. *hodgsoni*, *Se*. *khawi*, *Se*. *hivernus*, *Se*. *sylvatica*, *Ph*. *mascomai*, *Ph*. *barguesae*, *Ph*. *stantoni*, *Ph*. *betisi*, and *Gr*. *indica*. While two unidentified *Sergentomyia* sp. and one *Idiophlebotomus.* sp. were reported (Table 1). The most abundant species found in this study was *Se*. *anodontis* (44.72%, 165 samples) from Lampang and Chiang Rai provinces (69.17%, 83 samples). *Se*. *khawi* (60.29%, 41 samples) was the dominant species found in Songkhla province (Table 1; Fig. 1).

**Table 1:** Sand fly species composition, number of positive DNA samples tested for *Leishmania*, *Trypanosoma*, and *Bartonella* collected in the three studies sites in Thailand

Provinces	Sand fly species	No. of samples	Relative abundance (%)	Molecular detection of pathogens					
				*ITS1*-PCR	*SSU* rRNA-PCR	*gltA*-PCR	Prevalence rate (95% CI)		
				*Leishmania*	*Trypanosoma*	*Bartonella*	*Leishmania*	*Trypanosoma*	*Bartonella*
Lampang	*Sergentomyia anodontis*	165	44.72	0	0	2	0	0	1.21 (0.00–4.69)
(*n* = 369)	*Sergentomyia barraudi* s.l	62	16.80	0	0	0	0	0	0
	*Sergentomyia hodgsoni*	24	6.50	0	0	0	0	0	0
	*Sergentomyia* sp. 1	1	0.27	0	0	0	0	0	0
	*Phlebotomus mascomai*	105	28.46	0	7	0	0	6.67 (3.04–13.35)	0
	*Phlebotomus barguesae*	9	2.44	0	0	0	0	0	0
	*Phlebotomus stantoni*	1	0.27	0	0	0	0	0	0
	*Idiophlebotomus* sp.	2	0.54	0	0	0	0	0	0
Chiang Rai	*Sergentomyia anodontis*	83	69.17	0	2	12	0	2.41 (0.00–8.88)	14.46 (8.31–23.75)
(*n* = 120)	*Sergentomyia barraudi* s.l	16	13.33	0	0	1	0	0	6.25 (0.00–30.31)
	*Segentomyia khawi*	13	10.83	0	0	0	0	0	0
	*Sergentomyia hivernus*	5	4.17	0	0	0	0	0	0
	*Phlebotomus mascomai*	2	1.67	0	0	0	0	0	0
	*Grassomyia indica*	1	0.83	0	0	0	0	0	0
Songkhla	*Segentomyia khawi*	41	60.29	0	2	1	0	4.48 (00.00–17.01)	2.44 (0.00–13.74)
(*n* = 68)	*Sergentomyia barraudi* s.l	4	5.88	0	0	0	0	0	0
	*Sergentomyia anodontis*	2	2.94	0	0	0	0	0	0
	*Sergentomyia sylvatica*	2	2.94	0	0	0	0	0	0
	*Sergentomyia hivernus*	1	1.47	0	0	0	0	0	0
	*Sergentomyia* sp. 2	1	1.47	0	0	0	0	0	0
	*Phlebotomus betisi*	13	19.12	0	0	0	0	0	0
	*Phlebotomus stantoni*	4	5.88	0	0	0	0	0	0
Total		557		0	11	16	0	1.97 (1.06–3.55)	2.87 (1.74–4.65)

A total of 48 sand fly DNA samples were randomly selected to represent each morphologically identified species by *COI* sequencing. All of the selected DNA samples were successfully amplified with *COI* sequences for each sand fly species included in the study. For the K2P intraspecific genetic divergences result, two species had a highest maximum intraspecific divergence greater than 3%, including *Se*. *barraudi* (12.0%) and *Se*. *anodontis* (7.5%) (Supplementary 1: Table S1).

Additionally, 24 samples of *Se*. *hodgsoni* collected in Lampang, and 2 samples of *Se*. *sylvatica* from Songkhla provinces, demonstrating the BLASTn results of a randomly selected three *COI* sequences (PT15, PT89, and PT129) of *Se*. *hodgsoni* (89.21% similarity) and two *COI* sequence identities of *Se*. *sylvatica* (89.03% similarity), did not match with closely related species owing to missing data of these species in GenBank databases.

Two samples (PT341 and SD10) were morphologically identified only at the genus level and identified by *COI* sequencing (Supplementary 2: Fig. S1). BLASTn analysis of *Sergentomyia* sp.1 (PT341) shared 90.43% similarity with those sequences published in GenBank, while the result of BLASTn of *Sergentomyia* sp. 2 (SD10) showed a 99.29% similarity to an unidentified *Sergentomyia* sp. (accession number OK576213), previously reported in the Stun province in southern Thailand. However, the *COI* sequences of both samples were still only identified to the genus level as *Sergentomyia* species. Furthermore, two samples were morphologically identified into the genus *Idiophlebotomus*, and one successfully amplified *COI* sequence showed 96.27% similarity to *Id*. *teshi*.

**Fig. 2:**
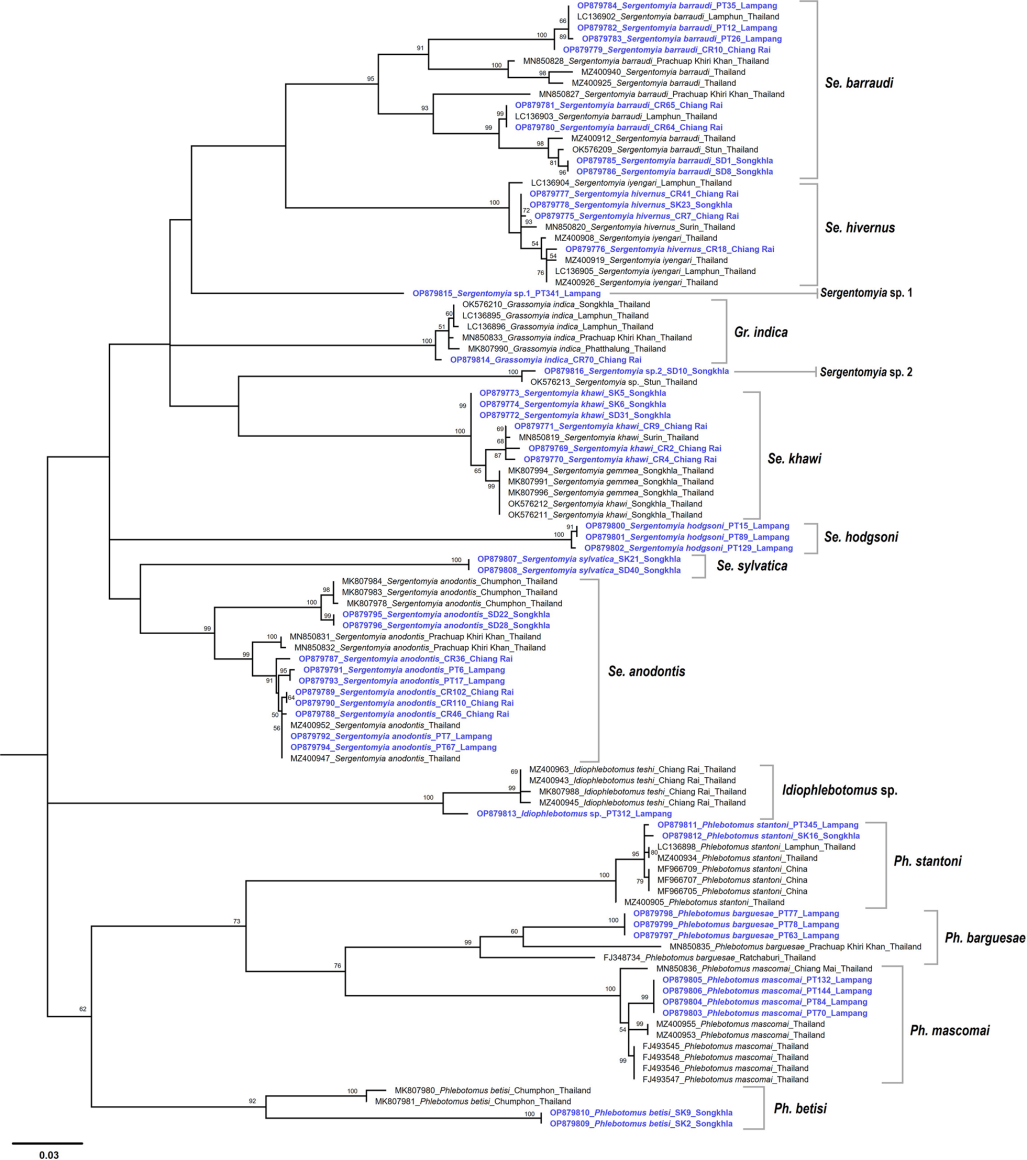
The phylogenetic relationship of sand flies was constructed from partial *COI* sequences of current sand fly samples and conspecific species obtained from the GenBank database. The tree was constructed using the ML analyses with the General Time Reversible + Gamma distribution (GTR + G) model (bootstrap 1000 times)

The phylogenetic construction based on the maximum likelihood method included 48 *COI* sequences from sand fly species processed during the present study, and 56 sequences of resembling sequences deposited in GenBank. In total, seven species were genetically classified into multiple lineages, including *Se*. *barraudi*, *Se*. *khawi*, *Se*. *anodontis*, *Se*. *hivernus*, *Ph*. *barguesae*, *Ph*. *betisi*, and *Ph*. *mascomai* (Fig. 2). Interestingly, a phylogenetic tree separated clusters of *Se*. *anodontis*, *Se*. *khawi*, and *Se*. *barraudi* samples, between the northern and southern parts of Thailand (Fig. 2). The *COI* sequences of the sand flies in this study were deposited in the GenBank database under accession number OP879769-OP879816.

### Detection of *Leishmania *and *Trypanosoma* DNA in sand flies

A total of 557 female sand flies were tested for *Leishmania* and *Trypanosoma* DNA on the basis of *ITS1* and *SSU* rRNA amplifications, respectively. The present study demonstrates that the TRY927F and TRY927R primer sets, targeted for the *SSU* rRNA gene, were able to successfully amplify all positive *Trypanosoma* DNA. Of these, 11 (1.97%) samples for *Trypanosoma* DNA, 7 *Trypanosoma* positives belonged to *Ph*. *mascomai* from Lampang, and 2 *Trypanosoma* positives were detected in *Se*. *anodontis* from Chiang Rai (Table 1). BLASTn analysis demonstrated a *Trypanosoma SSU* rRNA sequence length of approximately 937 bp in eight samples (PT82-10, PT84-62, PT96-49, PT132-17, PT144-69, PT154-73, PT158-28, and CR110-13), showing the ranged 99.89–100% sequence similarities with *T*. *noyesi* (accession number OP022194) that is available in the GenBank database. Furthermore, sample CR102-12 was consistent with the 930 bp length of *Trypanosoma SSU* rRNA sequence, the BLASTn result demonstrated a 99.89% similarity with *Trypanosoma* sp. (accession number MH989552), which was detected in sand flies from southern Thailand. From the detection of *Trypanosoma* in Songkhla, two samples (SK4-23 and SD33-33) were positively detected in *Se*. *khawi*. The result demonstrated that the *SSU* rRNA sequences were approximately 974 bp and 931 bp in length, sharing 99.74% (accession number MH989543) and 100% (accession number MH989552) identities for SK4-23 and SD33-33, with unidentified *Trypanosoma* found in previously reported sand flies in the Songkhla province of southern Thailand.

The maximum likelihood tree demonstrated that eight positive *Trypanosoma* were genetically classified to *T*. *noyesi* of the *T*. *cruzi* clade, which was detected in the sand flies from Thailand, as previously recorded in the GenBank database. Moreover, two positive *Trypanosoma* sp. (CR102-12 from Chiang Rai and SD33-33 from Songkhla) were distinctively classified into the An01 + An02/Frog2 lineage, while a positive *Trypanosoma* in Songkhla (SK4-23) was genetically clustered into the An04/Frog1 lineage of the anuran clades (Supplementary 3: Fig. S2). Using *ITS1*-PCR, *Leishmania* DNA was not detected in all sand flies tested in the present study. The *Trypanosoma SSU* rRNA sequences were assigned to the GenBank database with following accession numbers: OP861666-OP861676.

### Detection of *Bartonella* DNA in sand flies

All female sand flies were tested for *Bartonella* DNA by PCR on the basis of the *gltA* gene. The results showed that 16 (2.87%) samples tested positive for *Bartonella* DNA (Table 1). Of these, 13 samples were detected in *Se*. *anodontis* (12 samples) and *Se*. *barraudi* s.l. (1 sample) collected from Chiang Rai. Additionally, two samples of *Se*. *anodontis* (PT27-1 and PT250-17) infected with *Bartonella* sp. were trapped in Lampang while only one sample (SD39-8) was positive for *Bartonella* sp. in *Se*. *khawi* collected from Songkhla (Table 1). On the basis of BLASTn analysis, the *gltA* sequences of all samples were close to *Bartonella* sp. (accession number KP100345) in the GenBank database with a range of 97.08–98.67% identities. ML tree analysis demonstrated the presence of a new *Bartonella* sp. that was closely related to *Bartonella* sp. isolated from bats as previously reported in Vietnam and Thailand (Supplementary 4: Fig. S3). The *gltA* sequences were deposited in GenBank under the accession numbers OP903128-OP903143.

### Genetic diversity and haplotype analysis

**Fig. 3:**
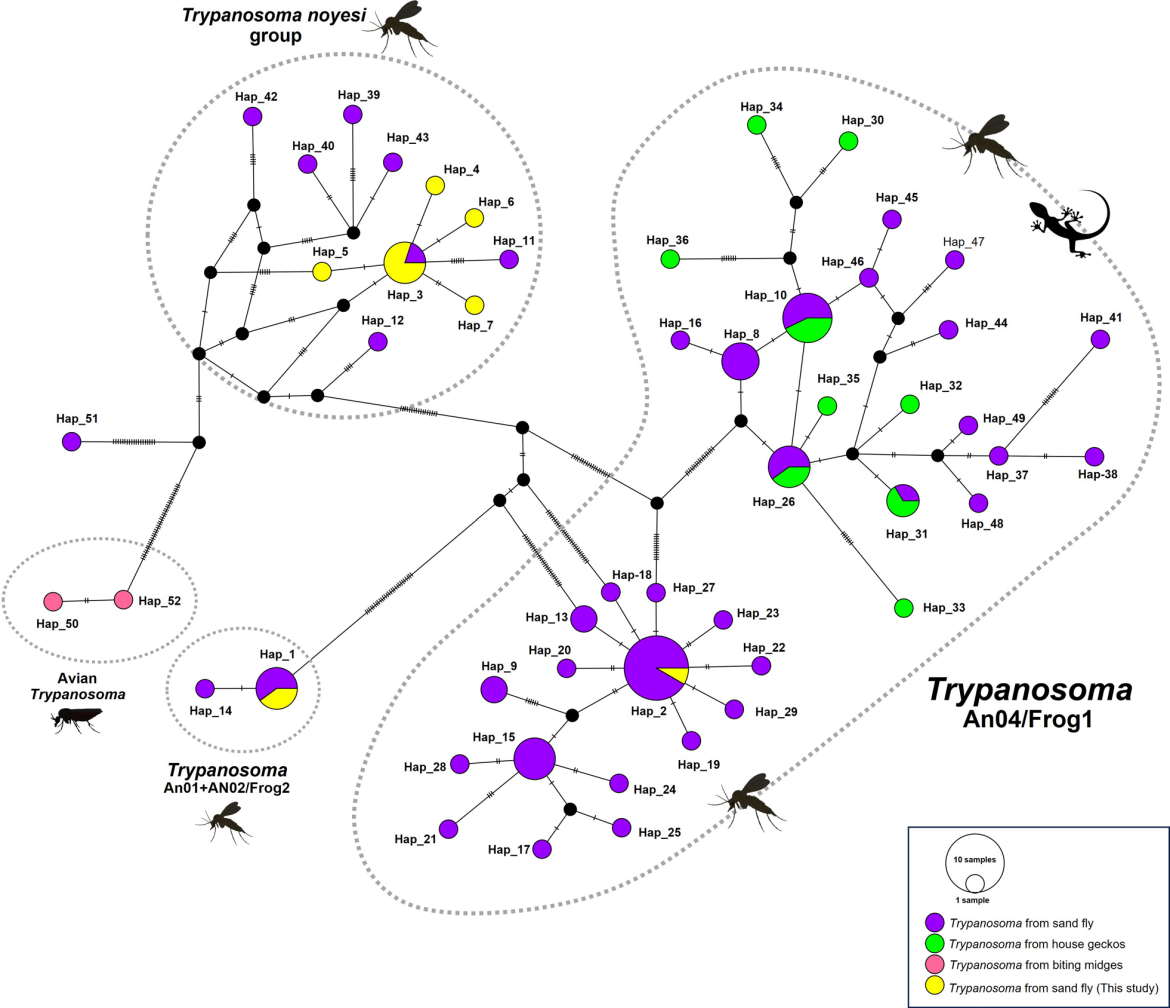
Haplotype network for *Trypanosoma SSU* rRNA sequences detected in sand flies collected from caves, compared with *Trypanosoma* sp. *SSU* rRNA sequences as previously detected in sand flies, house geckos, and biting midges in Thailand. The size of each circle is proportional to the number of sequences. Each bar in the branches represents a single nucleotide mutation

We investigated the genetic diversity and haplotype analysis of *Trypanosoma* sp. and *Bartonella* sp. based on *SSU* rRNA and *gltA* sequences. For *Trypanosoma* sp. isolates from Thai sand flies, 7 haplotypes out of 11 *Trypanosoma SSU* rRNA sequences demonstrated polymorphic sites (S) = 81, the mean number of nucleotide differences (*k*) = 26.40000, the haplotype diversity (Hd) = 0.873 ± 0.089, and the nucleotide diversity (*π*) = 0.03132 ± 0.01032 (Table 2). The *SSU* rRNA TCS network was generated for *Trypanosoma* spp. from the different hosts in Thailand (Fig. 3). The *Trypanosoma* haplotype network contained seven haplotypes, two sequences for Hap_1, four sequences for Hap_3, and one sequence each for Hap_2, Hap_4, Hap_5, Hap_6, and Hap_7. One *SSU* rRNA sequence was shared within Hap_2, which is the dominant haplotype belonging to sand flies in the An04/Frog1 lineage. Although two *SSU* rRNA sequences shared in Hap_1 were identified in sand flies of the An01 + An02/Frog2 lineage. The rest of the eight *SSU* rRNA sequences were formed differently of five haplotypes belonging to the *T*. *noyesi* group found in sand flies.

**Table 2:** Genetic diversity of *Trypanosoma* sp. and *Bartonella* sp. from sand flies in this study

Organisms (genes)	No. of sequences (*N*)	No. of haplotype (H)	No. of polymorphic sites (S)	Average number of nucleotide differences (k)	Haplotype diversity (Hd ± SD)	Nucleotide diversity (*π* ± SD)
*Trypanosoma* (*SSU* rRNA)	11	7	81	26.40000	0.873 ± 0.089	0.03132 ± 0.01032
*Bartonella* (*gltA*)	16	7	10	2.75833	0.85000 ± 0.054	0.00992 ± 0.00165

*SD* Standard Deviation

**Fig. 4:**
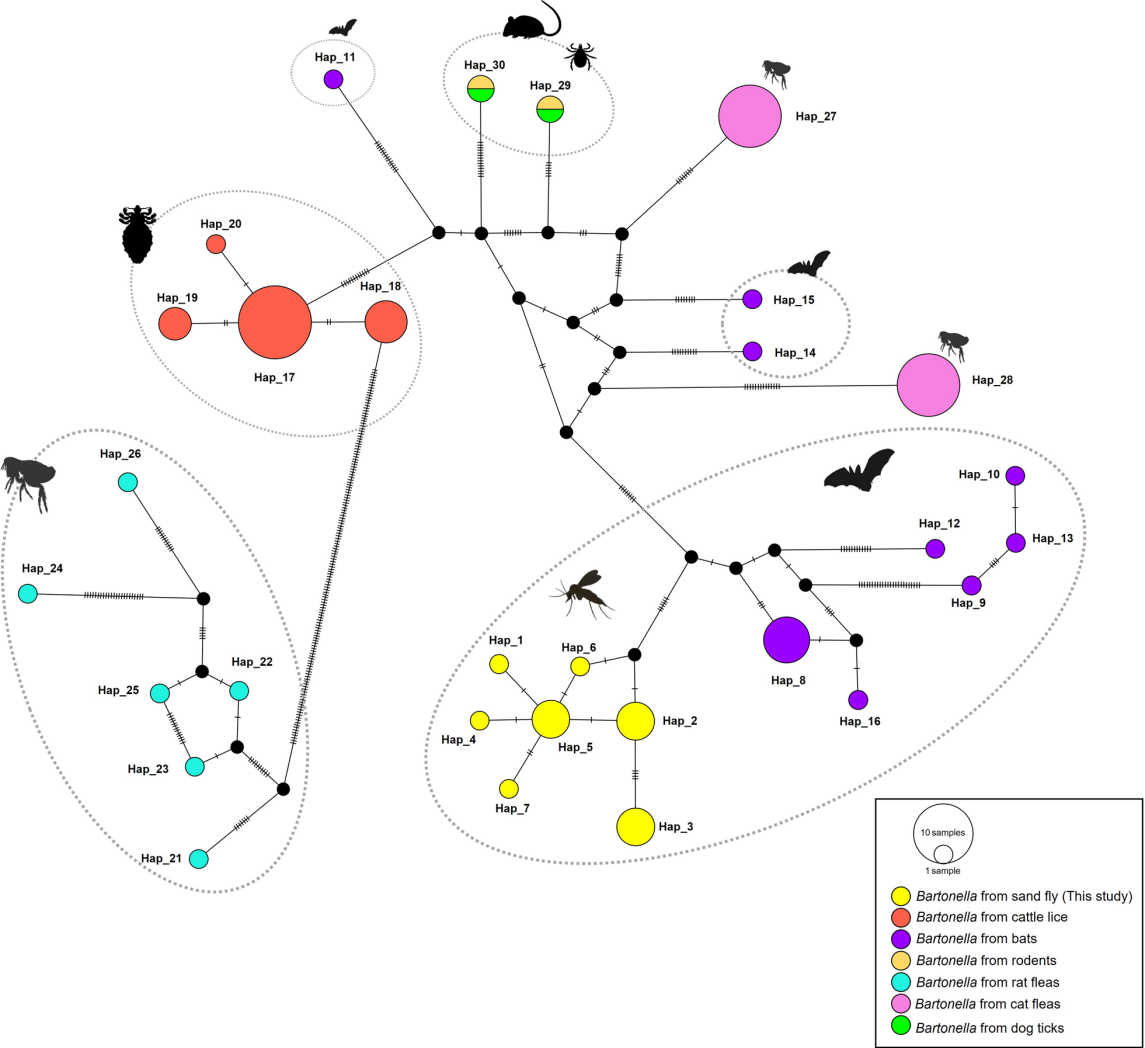
Haplotype network of partial *gltA* sequences of *Bartonella* sp. isolated from sand flies of this study and sequences of *Bartonella* sp. from different hosts including bats, rodents, cattle lice, rat fleas, cat fleas, and dog ticks reported in Thailand. The size of each circle is proportional to the number of sequences, and each circle represents a haplotype. The bar in the branches shows the number of mutations

The analysis of genetic diversity and *Bartonella* sp. using 16 *gltA* sequences showed seven haplotypes in all study sites (S = 10, k = 2.75833, Hd = 0.85000 ± 0.054, and *π* = 0.00992 ± 0.00165) (Table 2). The TCS haplotype network of *Bartonella* sp. demonstrated that 16 *Bartonella* sequences from this study contained 4 sequences for each Hap_2, Hap_3, and Hap_5, but 1 sequence for each Hap_1, Hap_4, Hap_6, and Hap_7. Furthermore, the *Bartonella* haplotype network in sand flies of this study was clustered separately from *Bartonella* sp. isolated from other hosts but closely related to *Bartonella* sp. from bats in Thailand (Fig. 4).

## Discussion

Despite Thailand having high levels of documentation of new species of phlebotomine sand flies, as well as published revisions, the taxonomy from the region of Southeast Asia is poorly studied. Taking into account that some types of samples are lost, or have never been deposited, the taxonomic revision of sand flies from Southeast Asia is taking time. To avoid misidentification, we have explained some of our taxonomic positions. Several survey studies were conducted in caves located in various regions of Thailand [4–7, 26, 52, 53]. In our study, 11 sand fly species were morphologically identified, namely, *Se*. *anodontis*, *Se*. *barraudi*, *Se*. *hodgsoni*, *Se*. *khawi*, *Se*. *hivernus*, *Se*. *sylvatica*, *Ph*. *mascomai*, *Ph*. *barguesae*, *Ph*. *stantoni*, *Ph*. *betisi*, and *Gr*. *indica*.

Among the molecularly identified sand fly species in this study, there is a possible presence of seven new species. These include *Se*. *barraudi*, *Se*. *khawi*, *Se*. *anodontis*, *Ph*. *barguesae*, *Ph*. *betisi*, *Ph*. *mascomai*, and *Idiophlebotomus* sp. Some sand fly species, such as *Ph*. *betisi*, showed a lower identity (89.70%) because this species had only two *COI* sequences published in the GenBank database, it was not enough to compare, and may have genetic diversity between our *COI* sequence and those sequences in the GenBank database. The *COI* sequences of *Se*. *hodgsoni*, *Se*. *sylvatica*, and two unidentified *Sergentomyia* sp. provided in this study are novel data for identification of these species in Thailand.

**Fig. 5:**
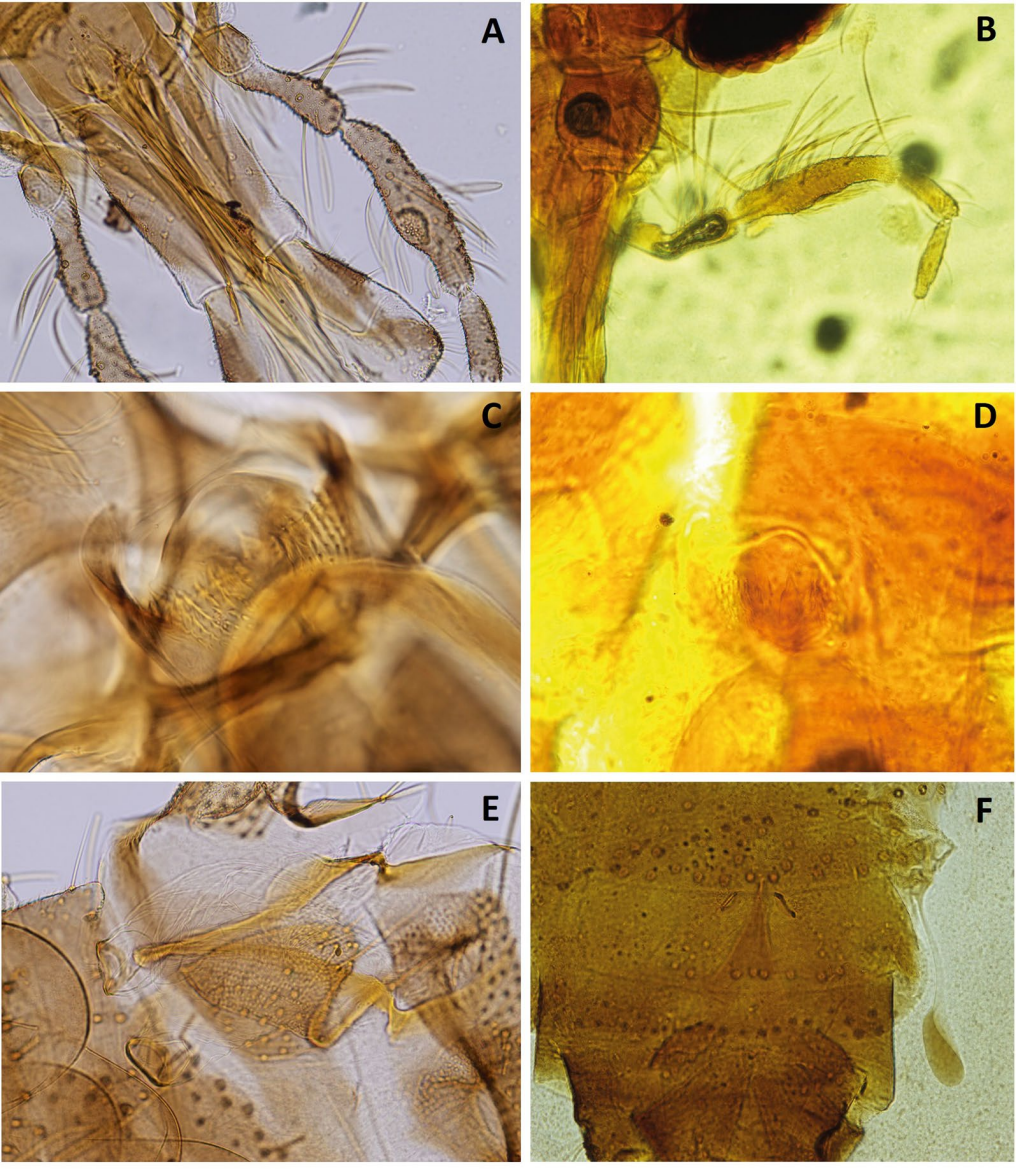
Comparison of *Idiophlebotomus* sp. found in this study (**A**, **C**, **E**) and *Id*. *teshi* holotype from Nepal (**B**, **D**, **F**): sensilla on third segment of palpi (**A**, **B**), cibarials teeth (**C**, **D**), and spermathecae (**E**, **F**)

The *Idiophlebotomus* caught in Lampang seems morphologically similar to *Id*. *teshi*, a species described from one female caught in Nepal (Fig. 5) [42]. The geographical distance of Nepal and Thailand, as well as probable climatic differences between the capture sites, lead us to the possibility that these sand flies could belong to different species. The morphological comparison of the holotype of *Id*. *teshi* with sample PT312 (Fig. 5), shows the appearance of the spermathecae is very similar, in particular because of the sclerification of the distal part. However, the cibarium, which shows an almost perfect alignment of the lateral teeth in the two samples, shows a V-structure in *Id*. *teshi* much more marked than in sample PT312. The distribution Newstead’s sensillae on the third segment of the palp is localized on a proximal process in *Id*. *teshi*, whereas they are on a distal process in PT312. This last sample has no antennae and therefore this character cannot be used to compare these individuals. Pending the description of the male from Nepal, as well as molecular data from this country, it seems to us more prudent not to propose a specific name for the PT312 sample.

For the species composition, *Se*. *anodontis* was the most abundant species trapped in the caves of Lampang and Chiang Rai in northern Thailand, and has been recorded as a cavernicolous species. This information is the same as the study conducted in the cave of Stun [54], Uthai Thani [55], and Chumphon provinces [17]. *Sergentomyia anodontis* is very easy to identify: females have a toothless cibarium except for a very characteristic large V-shaped structure. It is currently the only species that presents such an original cibarium, making its identification so easy. It is probably for the same reason that a few entomologists have taken much interest in this species and the minimal morphological variations it may include from one population to another. We noticed that some of them had more or less developed lateral vertical teeth. The molecular data, that very clearly separates samples from northern Thailand from those from the south of the country, are robust and supported by very high bootstrap values (99%). That leads us to believe that these two populations belong to two distinct species that should be compared by integrative taxonomy with samples from the type locality, located in Malaysia.

Although *Se*. *khawi* was the most recorded species in caves from Songkhla province in southern Thailand. This species of sand fly is reported as the potential vector for *L*. *martiniquensis* and is commonly found near the residence of patients with leishmaniasis [6, 7, 17, 26]. In contrast, *Se*. *khawi* was not found in any cave in the southern region as previously described by Phuphisut et al. [6] and Buatong et al. [7]. The time or season used in the study to collect the samples can affect the sand fly composition [54, 56]. Phylogenetic analysis on the basis of *COI* sequences of sand flies confirmed the morphological identification. What we called *Se*. *khawi* corresponds to female samples possessing 15–20 cibarial teeth and numerous vertical teeth generally arranged in two rows. *Se*. *khawi* belongs to what we may call the “*Se*. *iyengari*” group after the species described from southern India. This species had no vertical cibarial teeth but numerous records, probably all erroneous, have been reported from all over Southeast Asia [42]. Previously, there has been confusion between the morphological identification of *Se*. *gemmea* and *Se*. *khawi* [5]. This complex also includes other species whose synonymies may have been too hasty [57] such as *Se*. *malayensis* [58], *Se*. *hainanensis* [59], or *Se*. *taiwanensis* [60]. A comprehensive study of this complex including populations from India and Southeast Asia is needed to understand the relationships between these populations and revise the systematics of this group.

*Sergentomyia barraudi* has been described from males and females caught in the northeastern part of India by Sinton [61]. The type-specimen females exhibit smooth capsulated spermathecae and a cibarium with a forked sclerotized area and 40 palisadic teeth arranged along a straight line, this species is closely related to the *Se*. *barraudi* group. The taxonomy of this group is not resolved. As indicated by Vu et al. (2021), the *Se*. *barraudi* group should be further studied because of considerable heterogeneity in morphological characteristics, such as the number and distribution of teeth on the cibarium [62]. The molecular data of this study has shown to be an important variable and the taxonomy of this complex of species needs to be revised in future. There is no evidence for any segregation of the population within this complex, contrary to what we observed for *Se*. *anodontis* or *Se*. *khawi*, except the isolation of the samples from Songkhla.

*Phlebotomus barguesae* has been described from Thailand, more precisely from the province of Ratchaburi [64], which is located west of Bangkok at the same latitude. Molecular analysis shows that the three samples sequenced during the present study, which come from Lampang, are very clearly individualized from the two other *Ph*. *mascomai* included for comparison, including a type specimen (accession number FJ348734) and another (accession number MN850835) captured in the province of Phetchaburi, a little further south than the type specimen locality. Perhaps analogous to what we think about *Se*. *anodontis* among *Sergentomyia*. *Ph*. *barguesae* is very easy to differentiate from others of the subgenus *Euphlebotomus* owing to smooth spermathecae whose head is not separate from the body in the female. In the male, we observe in particular short and welded parameral sheaths. This perhaps overly simple identification does not encourage entomologists to examine the samples carefully. It is likely that an in-depth examination of several populations combining morphological and molecular approaches could potentially highlight differences between *Ph*. *barguesae* species, whose specific status could then be discussed.

The tree in Fig. 2 indicates that there is quite a marked variability in *Ph*. *mascomai*. However, this variability is less marked than that observed in *Ph*. *barguesae* owing to the branches bearing the various populations being relatively short. However, it is clear that the samples sequenced in this study are individualized and that a more in-depth study on this group would probably make it possible to better understand the taxonomy of this species and to detect possible close species.

The great length of the branches which carry, on the one hand the *Ph*. *betisi* of our study which come from the province of Songkhla, and those on the other hand, included by comparison (Fig. 2) raises differences which would deserve a further taxonomic study on a large number of populations and samples. Our *Ph*. *betisi* showed a low identity (89.70%) with the two comparison samples coming from the neighboring province of Chumphon (accession number MK807981 and MK807981). This is in agreement with their belonging to another species [65]. For reasons similar to those mentioned above for *Se*. *anodontis* or *Ph*. *barguesae*, *Ph*. *betisi* is the only *Phlebotomus* in this region whose males have a five-spined gonostyle and a simple paramere, and whose females have an annealed spermatheca and a head that is carried by a neck, which does not encourage the exclusion of a very detailed taxonomic study of these samples. Therefore, in the future, the progression of this study would be to conduct a comparative study of the type specimens species heterogeneity.

Furthermore, two *Sergentomyia* sand flies (*Se*. sp. 1 and *Se*. sp. 2) were identified at the genus level only by molecular identification on the basis of *COI* sequences. It seems likely that they belong to a new species. The study was limited to a single sample of each of the *Se*. sp. 1 and *Se*. sp. 2 samples, which may be insufficient for comparative analysis. The collection of additional samples for comparison would enable us to determine whether this is a new species of sand fly. However, a deep taxonomic investigation based on several samples must be carried out.

The intraspecific divergence analysis revealed high divergences in *Se*. *barraudi* (12.0%) and *Se*. *anodontis* (7.5%). Similarly, the phylogenetic analysis demonstrated a clear separation into three and two clades of *Se*. *barraudi* and *Se*. *anodontis*, respectively. These findings support the hypothesis that *Se*. *barraudi* and *Se*. *anodontis* constituted a cryptic species complex. The data are consistent with the findings of previous studies on cryptic species of *Se*. *barraudi* and *Se*. *anodontis* captured in a tourist cave from various regions of Thailand [6, 63]. Further taxonomic investigation and the consideration of different geographical structures are required to support the current findings for these species.

In the present study, natural infection with pathogens was carried out in sand flies collected from tourist caves located in three provinces in the northern (Lampang and Chiang Rai) and southern (Songkhla) parts of Thailand. All three provinces reported several cases of autochthonous leishmaniasis caused by *L*. *martiniquensis*. The role of sand flies as natural potential vectors for several pathogens, including *Leishmania*, *Trypanosoma* parasites, and *Bartonella* bacteria, was performed by PCR-based detection. We found 11 (1.97%; 11/557) positives for *Trypanosoma* DNA by using *SSU* rRNA*-*PCR in three sand fly species: *Ph*. *mascomai*, *Se*. *anodontis,*, and *Se*. *khawi*. A previous study in endemic and nonendemic areas of leishmaniasis in southern Thailand reported unidentified *Trypanosoma* and *Trypanosoma noyesi* DNA in various species of sand fly: *Se*. *khawi*, *Se*. *anodontis*, *Gr*. *indica*, *Id*. *asperulus*, *Ph*. *betisi* [17], and *Ph*. *stantoni* [26]. The phylogenetic relationship of the *SSU* rRNA sequences obtained in this study was divided into three groups: the first group was identified as *T*. *noyesi* within the *T*. *cruzi* clade, the second group was An04/Frog1, and the last group was An01 + An02/Frog 2, both groups were clustered in the anuran *Trypanosoma* clade.

In this study, *T*. *noyesi* was found in seven *Ph*. *mascomai* from Lampang and only one *Se*. *anodontis* from Chiang Rai provinces of northern Thailand, while the phylogenetic relationship was genetically clustered with *T*. *noyesi* of the *T*. *cruzi* clade. In contrast, a study by Khositharattanakool et al. has reported the presence of *T*. *noyesi* in two species of the genus *Idiophlebotomus*, namely *Id*. *longiforceps* and *Id.* *asperulus* [27]. The publication by Botero et al. has described the first novel species of *Trypanosoma noyesi* in an Australian marsupial (*Bettongia penicillate*) and also detected in tabanid and biting midges [66], additionally, *T*. *noyesi* was phylogenetically classified within the *T*. *cruzi* clade, which is a clade commonly found in bats [66, 67]. In a recent survey of *Trypanosoma* parasites in Thai bats on the basis of molecular detection, *T*. *noyesi* was identified from *Megaderma spasma* and phylogenetically clustered with *T*. *noyesi* from *Id*. *asperulus* sand flies as previously reported in Thailand [68]. The data suggest that sand flies may serve as a potential vector for this protozoan from host bats. Previously, a study of the detection of pathogens borne by sand flies has revealed *T*. *noyesi* DNA found in *Se*. *anodontis* and *Id*. *asperulus*, which was collected from a cave in the Chumphon, Trang, and Phatthalung provinces of the southern region of Thailand [17, 27]. On the contrary, all the tested sand fly samples collected from a cave in Songkhla province of southern Thailand were negative for *T*. *noyesi* DNA, while *T*. *noyesi* DNA was only found in the tested sand flies from the northern region of the country. However, this might be owing to the limitation of the sample sizes, and the difference between bat species and sand fly species that serve as a reservoir hosts and potential vectors of this protozoa species in the investigation area. The correlation of hosts, vectors, and parasites may still be unclear because we only detected DNA of *T*. *noyesi* in sand flies and did not investigate in bats. However, in an area of study for sand flies surveyed, we found many bats inside the cave. Bats are hypothesized to be reservoir hosts for *T*. *noyesi*, and sand flies may be a possible vector for the transmission of this parasite.

For other *Trypanosoma* detection, a *Trypanosoma* sp. from *Se*. *khawi* in Songkhla province is included in the *Trypanosoma* An04/Frog1 branch of the anuran clade. The anuran *Trypanosoma* has been described in several hosts, including toads, frogs, leeches, and invertebrates, such as sand flies [69–71]. In a previous study, the *Trypanosoma* parasite was isolated from *Ph*. *kazeruni* (accession number AB520638) sand flies in endemic areas of leishmaniasis from Pakistan and phylogenetically grouped with An04/Frog1, which was previously described as amphibian *Trypanosoma* (anuran clade) [72]. A study by Srisuton et al. reported that *Trypanosoma* sp. was detected in sand flies from Thailand, the unnamed *Trypanosoma* parasites within An04/Frog1, which is found in several sand fly species including *Se*. *khawi*, *Se*. *anodontis*, *Se*. *indica*, *Id*. *asperulus*, and *Ph*. *betisi* [17]. Two amphibian clades (An04/Frog1 and An01 + An02/Frog2) were detected in several sand fly species as previously reported by Preativatanyou et al. [21]. A recent study demonstrated *Trypanosoma* An01 + 02/Frog2 in *Se*. *khawi*, and *Trypanosoma* An04/Frog1 in *Se*. *khawi*, *Se*. *hivernus*, and *Gr*. *indica* from three regions of Thailand [27]. Furthermore, our two *Trypanosoma* sp. were genetically classified into the An01 + An02/Frog2 lineage found in each *Se*. *anodontis* from Chiang Rai (northern) and *Se*. *khawi* from Songkhla (southern). This study demonstrated that *Trypanosoma* sp. belongs to the An04/Frog1 and An01 + An02/Frog2 lineage of anuran trypanosomes. However, in addition to sand fly, anuran *Trypanosoma* DNA from An04/Frog1 was detected in flat-tailed house geckos (*Hemidactylus platyurus*) collected from an affected area of leishmaniasis in southern Thailand [73].

In addition to the study of *Trypanosoma* by the *SSU* rRNA gene, a study carried out by Buatong et al. [7] has revealed that the primers LeF and LeR targeted to the *ITS1* region for *Leishmania* detection is capable of amplifying *Trypanosoma* DNA in four species of sand fly: *Se*. *barraudi*, *Gr*. *indica*, *Se*. *khawi,* and *Id*. *asperulus* in southern Thailand; showing a phylogenetic relationship closely related to several species including *T*. *congolense*, *T*. *rangeli*, *T*. *lewisi*, *T*. *minasense*, *T*. *avium*, and unidentified *Trypanosoma* sp., which is close to *Trypanosoma* as reported in *Ph*. *stantoni* from southern Thailand [26]. Two primers have been suggested to amplify other Trypanosomatidae parasites [43]. We could not amplify *ITS1 Trypanosoma* by using this set of primers, and *Trypanosoma* sp. previously described by Phumee et al. [26] was not detected in the current investigation. However, the difference in species of *Trypanosoma* may be observed in our study. Furthermore, there is a lack of genetic marker sequences in the database to compare with the groups of trypanosomes or host origin by phylogenetic analysis. Several reports have suggested that the *SSU* rRNA, *18S* rRNA, and gGAPDH genes are the most common targeted markers to demonstrate phylogenetic relationships and classification of *Trypanosoma* parasites [44, 74, 75]. In our study, we used *SSU* rRNA sequences for phylogenetic analyses, and as a result were able to identify and classify *Trypanosoma* sp. with their phylogenetic relationships.

*Trypanosoma noyesi* is classified within the *T*. *cruzi* clade and has been identified in bats [68]. This parasite poses a significant risk to human and animal health. Nevertheless, there are no reported cases of *T*. *noyesi* being the causative agent of disease in humans. It is noteworthy that our study detected the presence of *T*. *noyesi* in cave-dwelling sand flies, which provides evidence that a bat trypanosome species may be a potential vector for the transmission of the parasites to humans or animals that enter the cave. Recently, microscopic evidence of live anuran trypanosomes, belonging to the AN04/Frog1 clade, was identified in sand flies collected from the residence of a patient with leishmaniasis in Songkhla province, southern Thailand [21, 27]. The presented evidence offers substantial support for the vectorial capacity of sand flies to transmit anuran parasites in amphibians and reptiles. Nevertheless, there is no evidence of anuran trypanosome infection in humans. It is therefore imperative that further epidemiological surveys of *Trypanosoma* in human and other animal hosts in the investigation areas be conducted to assess whether sand flies serve as a potential vector for the transmission of anuran trypanosomes.

In Thailand, several sand fly species and non-sand fly have been reported to be potential vectors of *L*. *martiniquensis* and *L*. *orientalis* in endemic areas of leishmaniasis in the southern and northern regions of Thailand [11, 17, 19, 20, 22, 23]. Recently, experiments on *Leishmania* subgenus *Mundinia* infections have been successful in the development and transmission of biting midges under laboratory conditions [76, 77]. The finding has suggested that biting midges could be important vectors, such as sand flies, for transmitting *L*. subgenus *Mundinia*. However, in the present study, we did not detect any *Leishmania* DNA in any collected sand flies. This study is correlated with previous studies of sand flies collected in the caves according to mentions by Panthawong et al. [78], Phuphisut et al. [6], and Buatong et al. [7]. It was assumed that the small sample size of specific sand fly species according to those described in Thailand. The present study was limited to the collection of sand flies in different seasons and varied habitat environments. Several pathogens rely on the presence of arthropod vectors for transmission, and the density of vectors is also highly sensitive to environmental factors, such as temperature and humidity [54, 79]. Further investigations of seasonal variation and additional habitats of sand fly collection are required.

In a previous study by Srisuton et al. [17], sand flies collected in a nonendemic area of leishmaniasis in Thailand also revealed negative results for the detection of *Leishmania*, but in an endemic area, there was a low DNA detection rate (5.41%), while a report by Sriwongpan et al. demonstrated an infection rate of *L*. *martiniquensis* in *Ph*. *stantoni* of 12.5% and *L*. *oriemtalis* in *Se*. *gemmea* of 10.9% [23]. Moreover, *Culicoides* biting midges are considered to be the potential vector of *Mundinia* species reported in Thailand, and the detection of *Leishmania* infection in a potential vector; *Culicoides* biting midges, demonstrated low DNA detection rate in natural conditions (2.83%) as previously reported by Sunantaraporn et al. [24]. A recent survey by Songumpai et al. revealed higher DNA detection rates of 21.2% from *Leishmania*-infected biting midges in the affected area than previous publications in sand flies, and the study in biting midges as described in northern Thailand, and also showed a lower DNA detection rate of 2.08% from *Leishmania*-infected biting midges in the nonaffected area in southern Thailand [25]. In future investigations, a larger number of sand flies and non-sand flies, such as *Culicoides* biting midges, from several collected areas should be investigated, and potential reservoir hosts are needed to provide strong information on leishmaniasis transmission in Thailand.

We detected 2.87% (16/557) of *Bartonella* DNA in different species of sand fly from three caves in Thailand, including *Se*. *anodontis*, *Se*. *barraudi*, and *Se*. *khawi*. Several publications have demonstrated *Bartonella* infection in New World sand flies, and sand fly species are demonstrated vectors of *Bartonella baciliformis* [28]. Moreover, other *Bartonella* have been detected in several species of American sand flies. Lozano-Sardaneta et al. revealed *Bartonella* sp. in *Lutzomyia* sp. collected from southern Mexico [80]. Zorrilla et al. found *Bartonella* spp. close to *B*. *bacilliformis* in *Nyssomyia whitmani*, *Pintomyia nevesi*, *Psychodopygus hirsuta*, *Pi*. *maranonensis*, and *Lutzomyia sherlocki* in Peru [81]. The study by Ulloa et al. detected the presence of *B*. *bacilliformis* DNA in 2 of 76 pools of *Pi*. *maranonensis* from Cajamarca, northern Peru [82]. *Bartonella* sp. is associated with rodents and humans, such as *B*. *grahamii*, *B*. *elizabethae*, and *B*. *rattimassiliensis* found in *Psathyromyia shannoni* and *Lu*. *cruciata* described by Lozano-Sardaneta et al. [83]. The similarity of our *Bartonella gltA* sequences and the phylogenetic relationship suggested that it could be related to *Bartonella* sp. detected in bats from Thailand and Vietnam. As previously described, bats are the natural reservoir host of several zoonotic infectious pathogens, including parasites, viruses, fungi, and bacteria, especially *Bartonella* bacteria [84]. Recent research by Poofery et al. has demonstrated zoonotic species of *B*. *ancashensis*, *B*. *henselae*, *B*. *bacilliformis*, and *B*. *australis*, as well as an unidentified *Bartonella* sp. in 24 bat species from Thailand [39], it was implied that bats serve as hosts of *Bartonella* bacteria, and it was assumed that sand flies feed on the blood from infected bats and obtain the bacterium. To clarify this hypothesis, blood meal identification in sand flies should be performed to provide data on the sand fly as a vector of zoonotic disease caused by bat-associated *Bartonella* species infection.

Previously, *Candidatus* Bartonella mayotimonensis has been identified as a potential pathogen in humans, and research has indicated that bats may serve as reservoir hosts for the bacteria [85]. It is conceivable that *Bartonella* sp. may be transmitted between bats and humans who enter the cave via exposure to feces. Nevertheless, the zoonotic transmission cycle of bat-associated Bartonellae in humans by sand flies remains incompletely understood. The data presented here reinforce the need for further investigation into the potential role of various sand flies in the transmission of *Bartonella* spp. to humans and animals.

*Bartonella bacilliformis* infection is considered the agent of human bartonellosis, namely Carrion’s disease or Oroya fever, which is transmitted by *Lu* *verrucarum* [33]. In Thailand, bartonellosis reported in humans was caused by several *Bartonella* species, including *Bartonella elizabethae*, *B*. *henselae*, *B*. *quintana*, *B*. *rattimassiliensis*, *B*. *tamiae*, *B*. *tribocorum*, and *B*. *vinsonii* [85]. However, bartonellosis caused by *Bartonella* associated with sand flies has never been reported. To the best of our knowledge, this is the first report of *Bartonella* bacteria detected in sand flies from Thailand.

In the current study, the partial *SSU* rRNA and *gltA* gene were used for genetic analysis, as these genes are the most common genetic markers for investigating the phylogenetic relationships and genetic diversity of the *Trypanosoma* parasite and *Bartonella* bacteria. We demonstrated seven haplotypes for each *Trypanosoma* and *Bartonella*. Genetic diversity on the basis of partial *SSU* rRNA and *gltA* genes showed a high haplotype diversity (Hd) and low nucleotide diversity (*π*) (Hd = 0.873 ± 0.089, *π* = 0.03132 ± 0.01032 for *Trypanosoma* sp. and Hd = 0.85000 ± 0.054, *π* = 0.00992 ± 0.00165 for *Bartonella* sp.). High haplotype diversity was observed in our *SSU* rRNA *Trypanosoma* sequences compared with previous reports, which are correlated with *Trypanosoma* detected in the sand fly (Hd = 1.000 ± 0.177, *π* = 0.013 ± 0.004) from Thailand [17, 68], *Trypanosoma* in *Be*. *penicillate* (Hd = 0.911 ± 0.077, *π* = 0.002 ± 0.0009) from Australia [66, 68], and *Trypanosoma* in bats (Hd = 0.603 ± 0.131, *π* = 0.045 ± 0.011) isolated from Thailand [68]. While the high haplotype diversity of *Bartonella* sp. observed in the *gltA* sequences is comparable to that demonstrated in bats (Hd = 0.890 ± 0.0081, *π* = 0.085 ± 0.013) in Thailand, as previously described [39], it was consistent with our sequence similarity to *Bartonella* found in bats. Unfortunately, the haplotype diversity and genetic diversity of *Bartonella* based on *gltA* sequences in the current study were not compared with that of *Bartonella* in sand flies in the previous study, owing to the lack of data and limitations of research studies of *Bartonella* in sand flies. A low *π* value among the *Trypanosoma SSU* rRNA and *Bartonella gltA* sequences in our study is congruent with the high similarity of these sequences of *Trypanosoma* sp. (99.74–100%) and *Bartonella* sp. (97.08–98.67%), suggesting that these genes seem to be highly conserved sequences. Furthermore, the higher haplotype diversity and low nucleotide diversity in *Trypanosoma* sp. and *Bartonella* sp. in sand flies indicate that these populations have recently diverged from one another [21]. It is of greater significance to note that the emergence of such genetic polymorphism may signify an evolutionary process by which the pathogens have successfully adapted to a diverse range of insect or reptile host species for *Trypanosoma* sp., and bat host species for *Bartonella* sp.

## Conclusions

The present study examined the species composition of sand flies collected from caves in the northern and southern regions of Thailand, and the natural infection and genetic diversity of *Trypanosoma* parasites and *Bartonella* bacteria in these insect vectors. Nevertheless, further epidemiological surveillance across different seasons, in diverse habitats, and across a range of host species is required to elucidate and refine the potential vectors of sand flies. This study is the first to report the presence of *Bartonella* DNA in sand flies. Furthermore, the vectorial role of sand flies or other vector species for the *Bartonella* bacteria, should be determined with the same *Bartonella* in blood from bats in different geographical regions in Thailand. It would be beneficial to gain a deeper understanding of the pathogens associated with sand flies and their potential to transmit to humans and animals. Sharing this information could be valuable in guiding future research and prevention strategies.

## Supplementary Information

**Additional file 1: Table S1.** Kimura 2-parameter intraspecific divergences of sand flies in the present study.

**Additional file 2: Figure S1.** Cibariums and spermathecae of *Segentomyia* sp. 1 and *Segentomyia* sp. 2.

**Additional file 3: Figure S2.** Maximum likelihood analysis of partial *SSU* rRNA sequences from *Trypanosoma* sp. based on the Kimura 2-parameter model with Gramma distributed (K2 + G). The bootstrap testing was conducted with 1000 replications.

**Additional file 4: Figure S3.** Phylogenetic analysis for *Bartonella* species based on partial *gltA* sequences. The ML tree was generated using the K2 + G model with 1000 bootstrap tests. *Brucella abortus* biovar 1 was used as an outgroup.

## Data Availability

The datasets used and analyzed during the present study are available from the corresponding author upon reasonable request. The sequence data obtained from this study have been submitted to the NCBI GenBank database (https://www.ncbi.nlm.nih.gov/genbank/) (accession nos. OP879769-OP879816 for sand fly species, OP861666-OP861676 for *Trypanosoma* sp., and OP903128-OP903143 for *Bartonella* sp.)
