# Supplementary material for: Cave-dwelling phlebotomine sand flies (Diptera: Psychodidae: Phlebotominae) in Thailand: population composition and pathogen detection of Bartonella and Trypanosoma
Source: Parasit Vectors. 2024 Dec 19;17:523. doi: 10.1186/s13071-024-06616-8 (PMC11661008; doi:10.1186/s13071-024-06616-8)
Supplement: Supplementary file 1 — Additional file 1: Table S1. Kimura 2-parameter intraspecific divergences of sand flies in the present study. [file 13071_2024_6616_MOESM1_ESM.pdf]

Table S1 Kimura 2-parameter intraspecific divergences of sand flies in the present study

Species	Number of sequences	K2P intraspecific genetic variation (min-max)
Phlebotomus barguesae	3	0.000
Phlebotomus betisi	2	0.000
Phlebotomus mascomai	4	0.000
Phlebotomus stantoni	2	0.005
Sergentomyia anodontis	10	0.000-0.075
Sergentomyia barraudi	8	0.000-0.120
Sergentomyia hivernus	4	0.000-0.021
Sergentomyia hodgsoni	3	0.000-0.005
Sergentomyia khawi	6	0.000-0.017
Sergentomyia sylvatica	2	0.000
Sergentomyia sp. 1	1	N/A
Sergentomyia sp. 2	1	N/A
Grassomyia indica	1	N/A
Idiophlebotomus sp.	1	N/A
