# Supplementary figures and images for: Cave-dwelling phlebotomine sand flies (Diptera: Psychodidae: Phlebotominae) in Thailand: population composition and pathogen detection of Bartonella and Trypanosoma
Source: Parasit Vectors. 2024 Dec 19;17:523. doi: 10.1186/s13071-024-06616-8 (PMC11661008; doi:10.1186/s13071-024-06616-8)

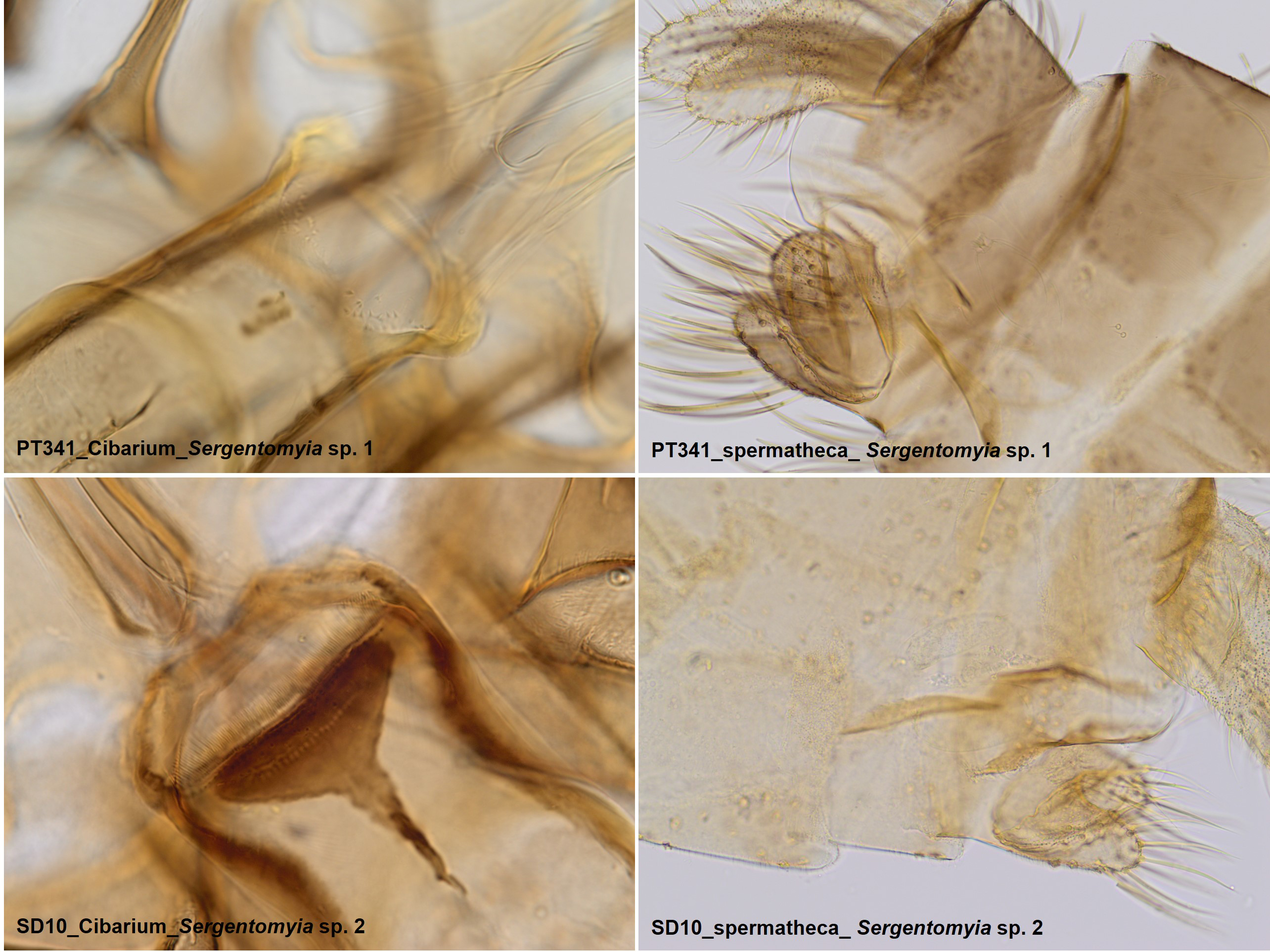

Supplement: Supplementary file 2 — Additional file 2: Figure S1. Cibariums and spermathecae of Segentomyia sp. 1 and Segentomyia sp. 2. [file 13071_2024_6616_MOESM2_ESM.tif]

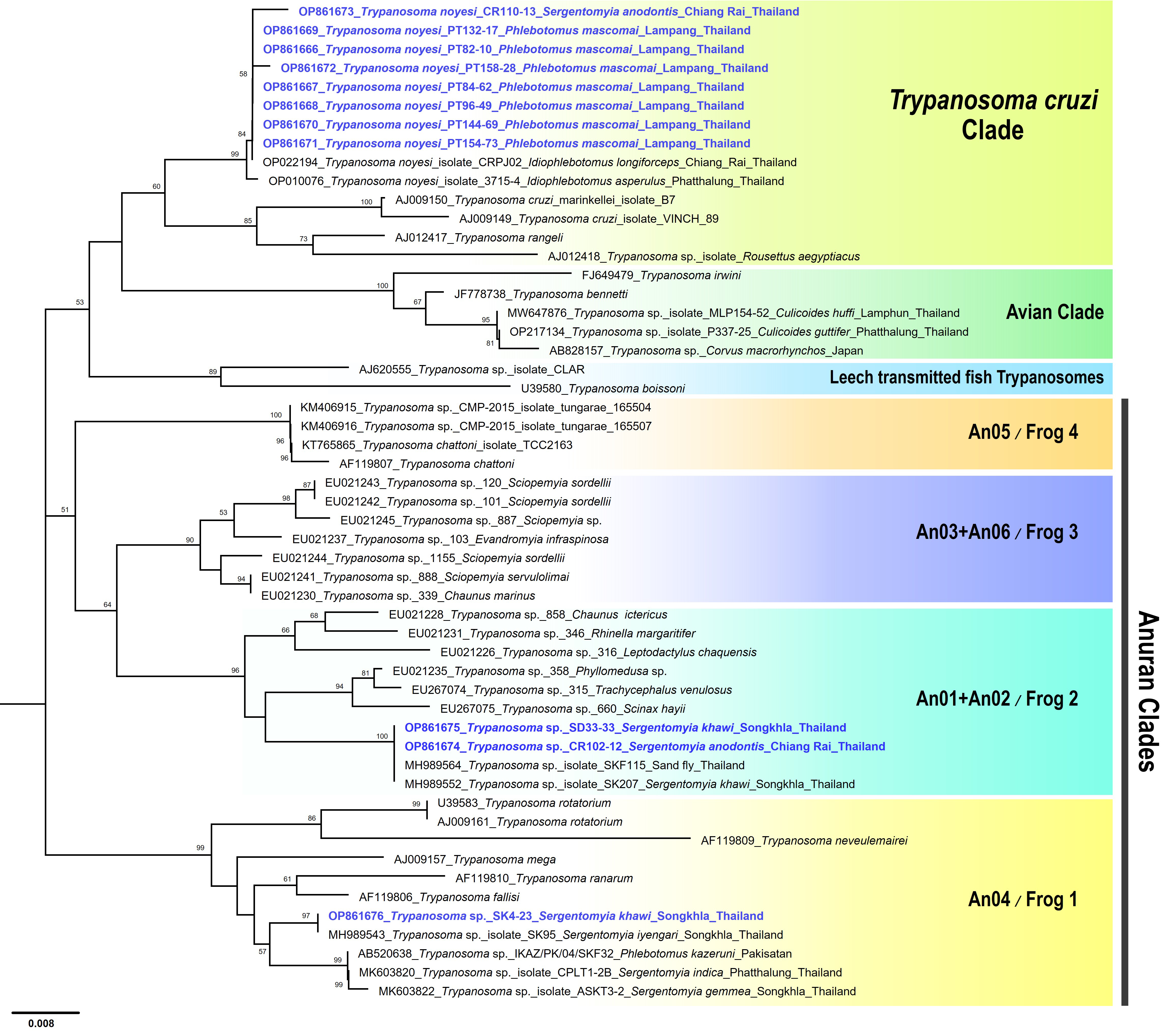

Supplement: Supplementary file 3 — Additional file 3: Figure S2. Maximum likelihood analysis of partial SSU rRNA sequences from Trypanosoma sp. based on the Kimura 2-parameter model with Gramma distributed (K2 + G). The bootstrap testing was conducted with 1000 replications. [file 13071_2024_6616_MOESM3_ESM.tif]

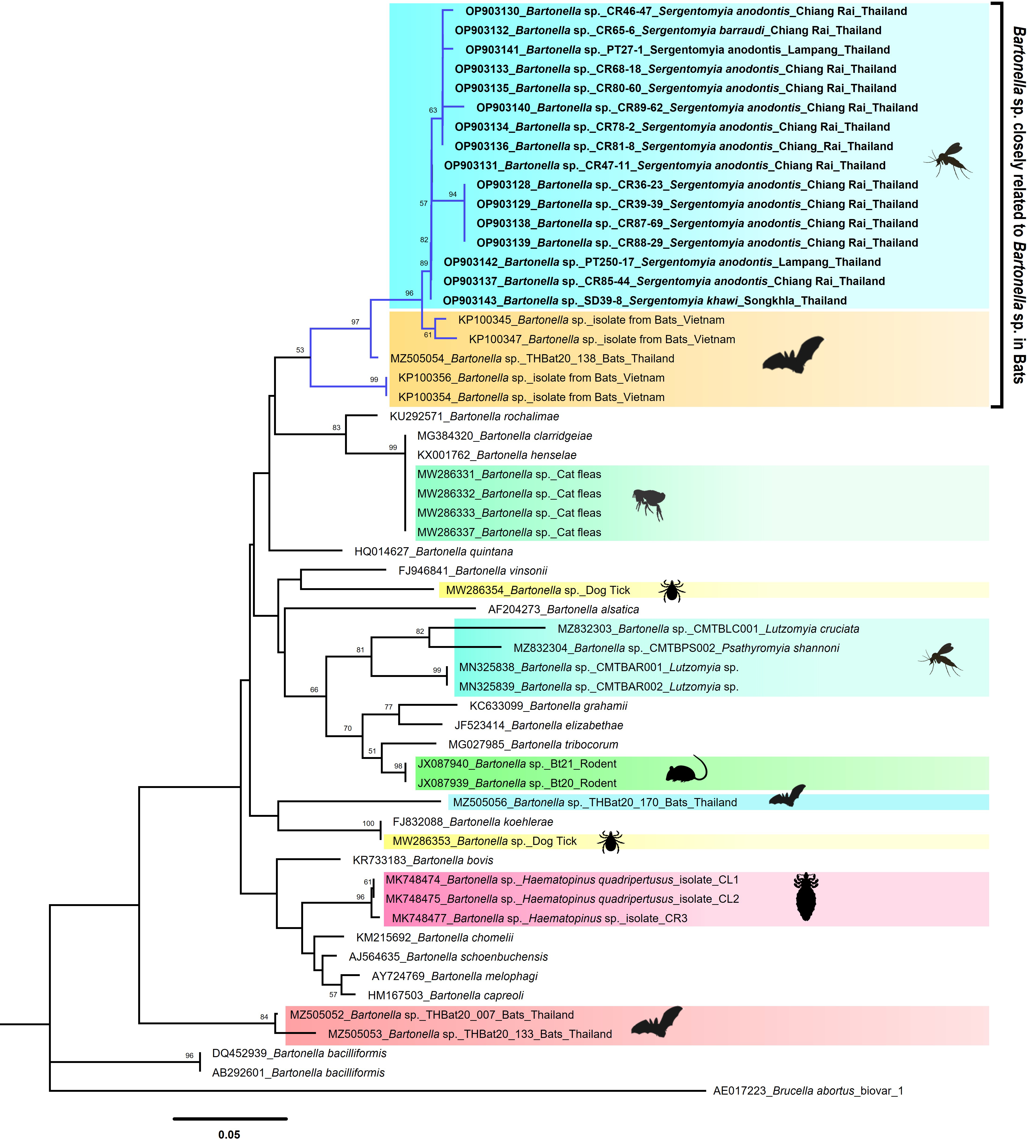

Supplement: Supplementary file 4 — Additional file 4: Figure S3. Phylogenetic analysis for Bartonella species based on partial gltA sequences. The ML tree was generated using the K2 + G model with 1000 bootstrap tests. Brucella abortus biovar 1 was used as an outgroup. [file 13071_2024_6616_MOESM4_ESM.tif]
